# Induction of galectin-1 expression by HTLV-I Tax and its impact on HTLV-I infectivity

**DOI:** 10.1186/1742-4690-5-105

**Published:** 2008-11-25

**Authors:** Sonia Gauthier, Isabelle Pelletier, Michel Ouellet, Amandine Vargas, Michel J Tremblay, Sachiko Sato, Benoit Barbeau

**Affiliations:** 1 Research Center in Infectious Diseases, CHUL Research Center, 2705 boul. Laurier; Ste-Foy, Québec, G1V 4G2, Canada; 2 Université du Québec à Montréal, Département des sciences biologiques, 2080 St-Urbain, Montréal, Québec, H2X 3X8, Canada

## Abstract

**Background:**

Cell-free Human T-cell Leukemia Virus type I (HTLV-I) virions are poorly infectious and cell-to-cell contact is often required to achieve infection. Other factors might thus importantly contribute in increasing infection by HTLV-I. Galectin-1 is a galactoside-binding lectin which is secreted by activated T lymphocytes. Several functions have been attributed to this protein including its capacity to increase cell-to-cell adhesion. Based on previous studies, we postulated that this protein could also accentuate HTLV-I infection.

**Results:**

Herein, we demonstrate that galectin-1 expression and release are higher in HTLV-I-infected T cells in comparison to uninfected T cells. Furthermore, galectin-1 expression was activated in various cell lines expressing the wild type viral Tax protein while this induction was minimal upon expression of NF-κB activation-defective TaxM22. Cotransfection of these Tax expression vectors with galectin-1 promoter-driven luciferase constructs confirmed that Tax upregulated galectin-1 promoter activity. However, a NF-κB-independent mechanism was strongly favoured in this induction of galectin-1 expression as no activation of the promoter was apparent in Jurkat cells treated with known NF-κB activators. Using HTLV-I envelope pseudotyped HIV-1 virions, galectin-1 was shown to increase infectivity. In addition, a co-culture assay with HTLV-I-infected cells also indicated an increase in cell fusion upon addition of galectin-1. This effect was not mediated by factors present in the supernatant of the HTLV-I-infected cells.

**Conclusion:**

These data suggest that HTLV-I Tax increases galectin-1 expression and that this modulation could play an important role in HTLV-I infection by stabilizing both cell-to-cell and virus-cell interactions.

## Background

Human T-cell Leukemia Virus type I (HTLV-I) is the etiological agent of adult T cell leukemia (ATL) and HTLV-I-associated myelopathy/tropical spastic paraparesis (HAM/TSP) [1-3]. It has been estimated that 20 million individuals are infected worldwide [4]. The *in vivo *target cells are mature CD4+CD45RO T lymphocytes and CD8+ T lymphocytes [5], although other cell types have been suggested to be potential target including lung epithelial cells, as recently demonstrated [6]. HTLV-I is transmitted between individuals by the transfer of infected lymphocytes and is thought to require repeated contacts as only one out of 1 × 10^5 ^to 1 × 10^6 ^viruses is infectious [7-9]. During viral transmission, a contact is established between an uninfected and an infected T cell by the interaction of the gp46 viral protein with its cellular receptor subsequently followed by the polarization of the infected cell cytoskeleton at the site of cell-to-cell contact and the accumulation of viruses at the cell junction [7]. GLUT-1 has been reported to be part of this receptor and to be involved in the first step of viral entry, although its exact role is still ill-defined [10,11]. Although the cellular ICAM-1 protein has been established as a potential inducer of microtubule reorganization, the viral Tax protein has also been shown to be active in this process [12,13].

Tax is the viral transactivator of HTLV-I allowing transcription through the three Tax-responsive elements (TRE1) present in the U3 region of the Long Terminal Repeat (LTR) [14-16]. This viral protein also promotes transcription of many cellular genes. To activate transcription, Tax does not bind directly to the different cellular and viral promoters but forms complexes with transcription factors, such as the cAMP Response Element Binding transcription factor (CREB). In uninfected cells, CREB phosphorylation leads to its interaction with CBP (CREB-binding protein) and the recruitment of the transcriptional machinery to CRE elements. In HTLV-I infected cells, Tax binds simultaneously to CBP and CREB and recruits the complex to viral TRE1 allowing constitutive LTR-dependent transcription [17]. Several studies have also provided detailed analysis on the mechanism of Tax-mediated activation of NF-κB by its association to IKK and upstream kinases [18]. Modulation of cellular genes by Tax has been extensively studied and has been shown to involve various transcription factors. In a previous study, using high-density gene arrays, 763 genes were shown to have differential gene expression profiles in HTLV-I-transformed and immortalized cell lines compared to peripheral blood mononuclear cells (PBMCs) [19]. One of the genes from which the expression was upregulated corresponded to the mammalian soluble β-galactoside-binding lectin, galectin-1 (LGALS1).

Galectins are a phylogenetically conserved family of proteins, present from invertebrates to mammals [20-22]. This family is constituted of at least 14 different galectins, most of which have an affinity for β-galactoside containing glycoconjugates, such as lactosamine residues [20,23]. The galectin family is further subdivided into three subfamilies: the prototype, the tandem repeat and the chimera groups [20]. Galectin-1 is a member of the prototype subfamily. While galectin-1 is primarily synthesized as a monomer that has one carbohydrate recognition domain (CRD), it also forms a dimer, which thus has the capacity to bind to two different β-galactoside-containing ligands. Galectin-1 is present in the cytoplasm of many cell types but can also be secreted [24-26]. Indeed, although nascent galectin-1 does not contain any signal sequence or hydrophobic domain necessary for usage of the secretory pathway, it has been well established that certain type of cells, such as activated T cells and thymus epithelial cells, secrete this lectin through a leaderless secretion pathway without compromising membrane integrity [22,24-28]. The expression of the galectin-1 gene is modulated during cellular differentiation and transformation [22,29]. Its expression is controlled by DNA methylation [30,31], known to restrict the access of transcription factors to binding sites [32]. The +1/+30 region of the galectin-1 gene is well preserved between different species [33] and the upstream (-57/-31) and downstream elements (+10/+57) of the initiation site account for the majority of the basal promoter activity [34]. However, little information is available on the transcription factor(s) involved in the modulation of the expression of this gene.

Being a dimer, galectin-1 could mediate cell-cell or cell-pathogen interactions. Indeed, our recent report suggests that galectin-1 stabilizes HIV-1 binding to its target, activating CD4+ T lymphocytes and therefore promoting HIV-1 infectivity [35,36]. Since an early report has suggested that HTLV-I-infected cells express galectin-1 [19] and HTLV-I infection requires cell-cell contact for several cell types, we investigated the pattern of expression of galectin-1 in infected cells and its possible impact on HTLV-I transmission. Our data show that Tax significantly induces transcription from the galectin-1 promoter in an NF-κB-, SRF- and CREB-independent manner. In fact, cell lines chronically infected by HTLV-I release more galectin-1 when compared to non-infected T cell lines. Furthermore, soluble galectin-1 increases HTLV-I cellular infection by HTLV-I gp46-pseudotyped HIV-1 virions. In addition, our data suggest that soluble galectin-1 enhances HTLV-I-mediated cell fusion between chronically infected cells and uninfected cells.

## Methods

### Cell culture and reagents

The following HTLV-I-infected cell lines were used in this study: C8166-45 [37], C91-PL [38], MJ [39], MT2 [40] and S1T [41]. The non-infected T cell lines, A2.01 [42], CEM-T4 [42], HSB-2 [43], Jurkat (clone E6.1) [44], Molt-4 [45], PM1 [46] and SupT1 [47] were also used. A2.01, CEM-T4, C8166-45, C91-PL, HSB-2, Molt-4, MT2 and PM1 were provided by the NIH AIDS Repository Reagent Program (Germantown, MD), while MJ and Jurkat E6.1 cells were provided by the American Type Culture Collection (ATCC) (Manassas, CA) and the S1T cell line was obtained from Dr. D. Branch (University of Toronto, Toronto, Canada). The 293T cell line [48] derives from human embryonic kidney cells and was obtained from the ATCC. PBMCs were isolated from healthy donors using Ficoll-Hypaque density gradient centrifugation. PBMCs were stimulated for 72 h with PHA-L (1 μg/ml) (Sigma-Aldrich, Oakville, Canada) and IL-2 (30 U/ml) and subsequently maintained in the presence of IL-2. All cell lines were maintained in complete medium (RPMI-1640 or DMEM) supplemented with 10% foetal bovine serum (Wisent, St-Jean-Baptiste de Rouville, Canada), L-glutamine (2 mM), penicillin (100 U/ml) and streptomycin (100 μg/ml) (Wisent, St-Jean-Baptiste de Rouville, Canada). The following reagent was obtained through the AIDS Research and Reference Reagent Program, Division AIDS, NIAID, NIH: Human rIL-2 from Dr. Maurice Gately, Hoffmann-La Roche Inc [49].

### Plasmids

Expression vectors for wild-type and mutant Tax proteins (i.e. Tax 703, Tax Δ3 and Tax M22) were obtained from Dr. K. Matsumoto (Osaka Red Cross Blood Center, Osaka, Japan) and cloned into phβPr.1neo under the control of the β-actin promoter [50]. The K30 proviral DNA was obtained from the NIH AIDS Repository Reagent Program. The pHTLV-Luc vector (kindly provided by Dr. W.C. Greene, University of California of San Francisco; San Francisco, CA) contains the luciferase gene under the control of HTLV-I LTR. The pNF-κB-Luc and pSRE-Luc luciferase expression vectors were purchased from Clontech (Mountain View CA). The pNL4.3Luc+Env-Vpr+ vector (kindly provided by Dr. N.R. Landau; The Salk Institute for Biological Studies, La Jolla, CA) encodes a complete HIV-1 genome in which the envelope gene has been inactivated and the luciferase gene inserted in the region coding for the Nef viral protein. The pSV HTLV-I env vector (kindly provided by Dr. R. Sutton, Baylor College of Medicine, Houston, TX) harbours the HTLV-I gp46 cDNA under the control of the SV40 promoter. The pActin-LacZ vector contains the β-galactosidase gene under the control of the actin promoter. The pLTRX-Luc construct was kindly provided by O. Schwartz (Unité d'oncologie virale, Institut Pasteur, Paris, France) and contains the HIV-1 LTR from the HIV-1 LAI strain positioned upstream of the luciferase reporter gene [51].

### Construction of the human galectin-1 promoter vector

A PCR-based approach was used to insert the luciferase gene under the control of the galectin-1 promoter. Genomic DNA was isolated from 293T cells with the QIAamp DNA Blood Mini Kit (QIAGEN, Mississauga, Canada). Two fragments of the galectin-1 promoter region (0.5 kb or 1.2 kb) were amplified from 200 ng of genomic DNA by PCR with the forward primers gal-0.5 kb (5'-GTTAAGTCAGTGGCCCTCTGCAG-3') or gal-1.2 kb (5'-CAGAGGAGATGTTAAGAGAGCAGAC-3') and the reverse primer gal-as1 (5'-CGCACCAGCTGTCAGAAGACTCC-3'). PCR amplifications were then performed in the presence of 0.2 mM dNTPs, 1 μM of each primer, 1 U of Vent polymerase (New England Biolab, Pickering, Canada) through 35 cycles (denaturing at 95°C for 1 min, annealing at 63°C for 1 min and polymerizing at 72°C for 1 min). The PCR products were purified with the QIAquick PCR purification kit (Qiagen, Mississauga, Canada) and ligated into the pBluescript SK (pBSK) vector in SmaI. Positive clones were sequenced and compared to the human galectin-1 promoter sequence (Genbank Accession no [Z83844.5]). The 0.5 kb and 1.2 kb galectin-1 promoter fragments were cut out of pBSK with SacI and NdeI enzymes and ligated into pGL3-Basic (Promega; Neapean, Canada) digested by SacI and SmaI.

### Preparation of galectin-1

Recombinant human galectin-1 was purified as previously described [35]. Purified galectin-1 was passed through Detoxi-gel endotoxin-removing gels (Pierce; Rockford, IL). The activity of galectin-1 to bind to glycan and to cross-link neighbouring cells was weekly tested by performing a hemagglutination assay with concentrations ranging from 1 to 4 μM.

### RT-PCR

Total RNA from A2.01, HSB-2, Jurkat (clone E6.1), Molt-4, CEM-T4, PM1, Sup T1, C8166-45, C91-PL, MJ, MT2 and S1T cell lines or from transfected 293T cells was extracted with the TRIzol reagent (Invitrogen; Burlington, Canada). Extracted RNA (5 μg) was then reverse transcripted with the M-MLV reverse transcriptase (1 U) (Invitrogen; Burlington, Canada) and oligo dT primers. Next, PCR amplification was performed on the resulting cDNA with primers act-s (5'-CGTGACATTAAGGAGAAGCTGTGC-3') and act-as (5'-TCTAGGAGGAGCAATGATCTTGAT-3') for β-actin mRNA; gal-s (5'-GACTCAATCATGGCTTGTGGTCTG-3') and gal-as (5'-GCTGATTTCAGTCAAAGGCCACAC-3') for galectin-1 mRNA; or tax-s (5'-ATGGCCCACTTCCCAGGGTTTGGAC-3') and tax-as (5'-TCAGACTTCTGTTTCGAGGAAATG-3') for Tax mRNA. PCR amplifications were performed in the presence of 0.2 mM dNTPs, 1 μM of each primer, 1 U Vent polymerase and 30 amplification cycles (denaturation at 95°C for 1 min, annealing at 55°C for galectin, 58°C for β-actin and 65°C for Tax for 1 min and polymerization at 72°C for 1 min). The PCR products were then migrated on a 1.5% agarose gel.

### Real-time RT-PCR

RNA was first isolated from 293T transfected cells, by the RNeasy^® ^Plus mini Kit (Qiagen, Mississauga, ON, Canada) according to the manufacturer's instructions. Real-time RT-PCR reactions were then performed in the presence of each specific primer. Briefly, RNA (5 μg) was reverse transcripted with the M-MLV reverse transcriptase (1 U) (Invitrogen) and oligo dT primers. PCR reactions were then initiated in a final volume of 10 μl containing 1 μl of cDNA, 0.5 μM of each primer, and 1× reaction mix, including Taq DNA polymerase, the reaction buffer, and SYBR green (SYBR^® ^Premix Ex Taq™ Perfect Real Time, Fisher Scientific Canada, Montréal, Canada). All primer sequences were generated using the Light Cycler Probe Design Software 2.0 (Roche, Basel, Switzerland) and checked for specificity using GenBank Blast analysis. The galectin-1 primers were the following: 5'-GACTCAATCATGGCTTGTGGTCTG-3' (reverse) and 5'-GCTGATTTCAGTCAAAGGCCACAC-3' (forward). In all PCR reactions, negative controls consisting of a RT-like reaction step with no added reverse transcriptase in addition to a blank sample were carried out and showed no PCR amplification (data not shown). Thermal cycling for quantification of both transcripts was initiated with a denaturation step of 95°C for 10 seconds, followed by 50 cycles (denaturation at 94°C for 3 seconds, 57°C for annealing during 15 seconds, and elongation at 72°C for 12 seconds). Amplification of the human HPRT-1 (Hypoxanthine Phosphoribosyl Transferase 1) cDNA with forward and reverse primers (5'-AAGCTTGCGACCTTGACC-3' and 5'-GACCAGTCAACAGGGGACATAA-3', respectively) was used as a reference gene for normalisation. To verify the amplification of each single product with its suitable melting temperature, and to provide an accurate quantification with the Rel Quant Software, dissociation curves were run for all reactions and amplified products were visualized by electrophoresis on a 1.5% agarose gel.

### Transient transfections

Jurkat, CEM-T4 and SupT1 cells (1 × 10^7^) were transiently transfected by electroporation as previously described [52]. Briefly, cells were electroporated with 15–20 μg of DNA in complete medium containing 10 μg/ml DEAE-DEXTRAN in a 0.4 cm electroporation cuvette with the Bio-Rad Gene Pulser II system (250 V, 950 μF). In transfection experiments assessing NF-κB activation, 24 hours after transfection, cells were either untreated or treated with PMA (20 ng/ml) or TNF-α (10 ng/ml) (Sigma-Aldrich, St-Louis MO) for a period of 8 hours. For the Sup T1 cell line, DMSO was also added at a final concentration of 1.25%. For certain experiments, extracted RNA were analysed by RT-PCR, while luciferase activity was evaluated in other transfection experiments as previously described [53]. In these latter experiments, β-galactosidase activity was also measured through the Galacto-Light™ commercial kit (Applied Biosystems, Bedford, MA) according to the manufacturer's protocol. Experiments were conducted in triplicates and both luciferase and β-galactosidase activities are represented as the average value +/- standard deviation. Transfection of 293T cells with the various Tax expression vectors (40 μg) were performed as previously described [54].

### Quantification of extracellular galectin-1 levels

A2.01, HSB-2, Jurkat (clone E6.1), Molt-4, PM1, CEM-T4, SupT1, C8166-45, C91-PL, MJ, MT2 and S1T cell lines were seeded at 5 × 10^5 ^cells/ml, and incubated for 48 hours. The supernatants were passed through a 0.22 μm filter, and lysed with a 5× disruption buffer (PBS 1×, 0.05% Tween-20, 2.5% Triton X-100 and 1% Trypan blue). Galectin-1 concentration was determined by an in house ELISA assay specific for galectin-1.

### Virus production and infection assay

HIV-1-based viruses pseudotyped with the HTLV-I envelope protein were prepared as previously described [54]. Briefly, 293T cells were cotransfected with 13 μg of the envelope-defective luciferase-expressing HIV-1 proviral clone pNL4.3L+E-Vpr+ and 26 μg of pSV HTLV-I env by calcium phosphate coprecipitation. The cells were washed with PBS 1× 16 hours after transfection and incubated another 24 hours. Supernatants were then filtered through a 0.22 μm-pore-size filter to remove cells and cellular debris. Viral preparations were stored at -85°C until needed. Virus particles were titrated through the use of a sandwich ELISA specific for the HIV-1 p24 capsid protein [55]. Pseudotyped virions were subsequently used in infection experiments of Jurkat and PBMCs. Cells were initially incubated with various concentrations of galectin-1 (ranging from 0 to 4 μM) for 30 minutes in the absence or presence of 50 mM lactose and then infected with luciferase-encoding HTLV-I env-pseudotyped viruses (10 ng of p24 per 1 × 10^5 ^cells) for 48 hours at 37°C before lysis. In certain experiments, 24 hours after transfection, TNF-α was added at a concentration of 10 ng/ml. Luciferase activity was next measured as previously described [53]. Experiments were conducted in triplicates and luciferase activity represents the average value +/- standard deviation.

### Co-culture assays

Jurkat cells were transfected with pHTLV-Luc by electroporation as described above. HTLV-I-infected C91-PL cells (1 × 10^5^) were then added to an equal number of transfected Jurkat cells in a flat-bottom 96-well plate. Galectin-1 was added in various concentrations (ranging from 0 to 4 μM) in the absence or presence of 50 mM lactose for 24 hours at 37°C before lysis and quantification of luciferase activity. As a control, transfected cells were similarly incubated with supernatant of C91-PL cells harvested after a 24 hour incubation at a concentration of 1 × 10^6 ^cells/ml and filtered through a 0.22 μM filter. Values are expressed as the average luciferase activity +/- standard deviation calculated from triplicates.

### Statistical analyses

Statistical analyses were carried out according to the methods outlined in Zar (1984) [56]. Homoscedasticity were determined using F_max_. When homoscedasticity assumptions were met, means were compared using Student's t test, or a single factor ANOVA followed by Dunnett's multiple comparisons when more that two means were considered. When homoscedasticity assumptions were not met, means were compared using a Kruskal-Wallis single factor ANOVA followed by Dunnett's multiple comparisons when more than two means were considered. *P *values of less than 0.05 were deemed statistically significant, whereas *p *values lower than 0.01 were considered highly significant. Computations were carried out using GraphPad PRISM version 3.03 statistical software.

## Results

### Galectin-1 is more strongly expressed in HTLV-I-infected T cells than in non-infected T cells

**Figure 1:**
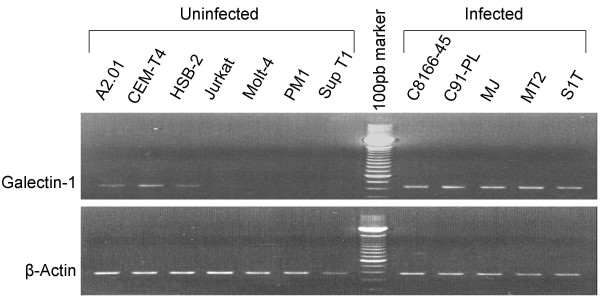
**Comparative analysis of galectin-1 expression in different uninfected T cell lines and HTLV-I chronically-infected cell lines**. Galectin-1 mRNA levels were measured by RT-PCR analyses on total RNA isolated from non-infected (A2.01, CEM-T4, HSB-2, JurkatE6.1, Molt-4, PM1, and Sup T1) and chronically HTLV-I-infected cells (C8166-45, C91-PL, MJ, MT2 and S1T). PCR products were separated by electrophoresis on 1.5% agarose gels. Expression of β-actin mRNA served as an internal control for normalization.

Previous studies have suggested that expression of various genes are positively modulated in HTLV-I-infected cells [19,57]. In order to determine whether galectin-1 expression is indeed altered in HTLV-I-infected cells, RT-PCR experiments were performed to compare the level of galectin-1 gene expression between non infected human T cells and HTLV-I-infected human T cells. Sequence-specific primers were derived from two different exons to insure that amplified products were derived from cDNA and not contaminating genomic DNA. As presented in Figure 1, results showed that galectin-1 was expressed in all HTLV-I-infected cell lines studied in contrast to non-infected T cell lines in which galectin-1 mRNA expression was either undetectable or slightly expressed. These results hence suggested a possible association between HTLV-I infection of T cells and increased expression of galectin-1.

### Tax induces galectin-1 expression

**Figure 2:**
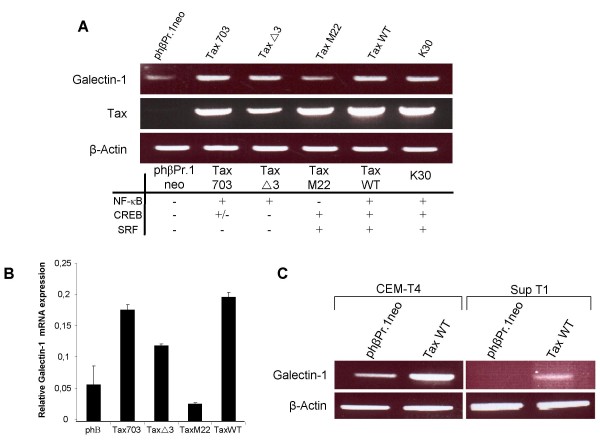
**Analysis of galectin-1 expression in WT and mutant Tax-expressing cells**. **A,B**. 293T cells were transfected with 40 μg of the control vector phβPr.1neo, Tax expression vectors (Tax 703, TaxΔ3, Tax M22, and Tax WT) or full-length proviral DNA K30 clone. RT-PCR analyses for galectin-1, Tax and β-actin RNA levels (**A**) and real-time RT-PCR for galectin-1 RNA levels (**B**) were conducted on RNA from each transfected conditions. The activated transcription factors for each Tax expression vectors are indicated below panel **A**. **C**. CEM-T4 and Sup T1 cell lines were transfected with 20 μg of the control vector pHβPr.1neo or Tax WT expression vector. Total RNA was analyzed by RT-PCR for galectin-1 and β-actin RNA levels. PCR products were separated by electrophoresis on 1.5% agarose gels.

As some of the tested HTLV-I-infected cells have been reported to only express the viral Tax protein, we then looked if Tax expression indeed could modulate galectin mRNA levels. 293T cells were transfected with either a vector containing a complete HTLV-I proviral genome (i.e. K30), or expression vectors coding for Tax WT or Tax mutants defective in their ability to activate transcription factors NF-κB, SRF and/or CREB. Galectin-1 expression was then analyzed by RT-PCR. As shown in Figure 2A, transfection of the K30 proviral DNA led to an induction in the expression of galectin-1. In addition, comparable induced levels of galectin-1 mRNA were observed in 293T cells expressing wild-type Tax and both Tax mutants defective for CREB and SRF activation (Tax 703 and Tax Δ3). In contrast, cells that were transfected with the Tax M22 (deficient in NF-κB activation) expression vector did not demonstrate a significant difference in galectin-1 mRNA levels when compared to cells transfected with the control vector (Figure 2A). As RT-PCR experiments further show that cells expressed similar levels of Tax, this difference in upregulation of galectin-1 mRNA level was not due to differences in the expression level of the different Tax proteins in transfected 293T cells. In order to confirm these results, RNA from 293T cells transfected with the various Tax expression vectors were quantitatively analysed for galectin-1 expression by real-time RT-PCR. Results presented in Figure 2B again revealed an important decrease in Tax M22-mediated activation of galectin-1 expression while other Tax mutants demonstrated a comparable upregulation to the one measured with wild-type Tax.

Next, RT-PCR analyses were performed in a more representative context, i.e T cell lines. Hence, the wild-type Tax expression vector was transfected in CEM-T4 and SupT1 T cell lines and analysed by RT-PCR for galectin-1 expression. As denoted in Figure 2C, Tax expression indeed increased the expression of galectin-1 in both T cell lines.

As the data suggest that HTLV-I Tax induces the expression of galectin-1 in non-T and T cell lines, it is likely that Tax plays a role in the modulation of galectin-1 mRNA levels in HTLV-I-infected cell lines.

### Tax induces transcription from the galectin-1 promoter

**Figure 3:**
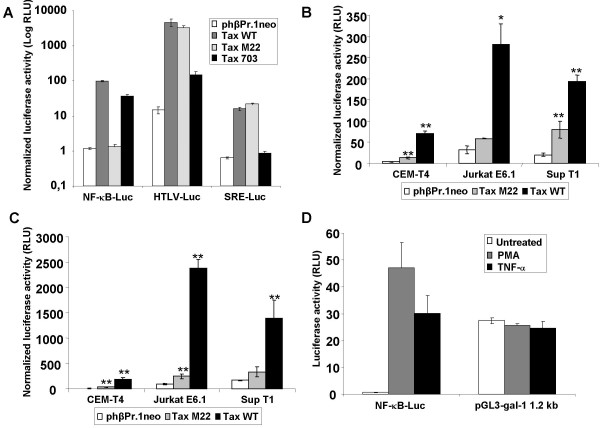
**Activation of the galectin-1 promoter by Tax expression in transfected T cell lines**. **A**. Jurkat cells were transfected with either pNF-κB-Luc, pHTLV-Luc or pSRE-Luc (7.5 μg) along with pHβPr.1neo (control vector) or expression vectors for Tax WT, Tax M22 or Tax 703 (7.5 μg) and pActin-LacZ (5 μg). **B,C**. Jurkat, CEM-T4 and Sup T1 T cell lines were co-transfected with pHβPr.1neo (control vector) or expression vectors for Tax WT or Tax M22 (7.5 μg), the galectin-1 promoter reporter constructs pGL3-gal-1 0.5 kb (**B**) or pGL3-gal-1 1.2 kb (**C**) (7.5 μg) and pActin-LacZ (5 μg). **D**. Jurkat cells were transfected with pNF-κB-Luc or pGL3-gal-1 1.2 kb (15 μg). After transfection (24 hours), cells were either left untreated or stimulated with PMA or TNF-α for 8 hours. Luciferase and β-galactosidase activities were determined 48 hours after transfection as described in Materials and Methods. In panels **A**, **B **and **C**, luciferase activity was normalized on the basis of the β-galactosidase activity. The results represent the mean of three independent transfections +/- standard deviations (*p < 0.05; **p < 0.01).

To determine whether the effect of Tax on galectin-1 expression resulted from direct activation of transcription from the galectin-1 promoter, two different luciferase-encoding vectors driven by the human galectin-1 promoter were constructed. Two fragments of 0.5 kbp and 1.2 kbp containing the transcription initiation site deduced from sequence homology with the mouse galectin-1 gene were derived from the human galectin-1 promoter region. Both fragments were cloned upstream of the luciferase reporter gene of the pGL3-Basic vector. Before determining the effect of Tax on these constructs, the Tax M22 expression vector was first tested in the context of Jurkat cells to see if it was specifically deficient in activating NF-κB (Figure 3A). These results indeed confirmed previous studies in Jurkat cells: Tax M22 was only defective in activating NF-κB unlike Tax 703, which was comparable to wild-type Tax for NF-κB activation but greatly affected in its capacity to activate both SRF and CREB (the latter being tested with the HTLV-I LTR-driven reporter construct mainly responsive to CREB activation). As Tax M22 was behaving as expected in the Jurkat T cell line, the two galectin-1 promoter constructs were next cotransfected with Tax WT or Tax M22 expression vectors along with pActin-LacZ into CEM-T4, Jurkat E6.1 and SupT1 T cell lines and promoter activity was then evaluated by luciferase activity after normalisation (Figure 3B, C). When compared to cells transfected with the control vector, the 0.5 kb galectin-1 promoter construct demonstrated an increase of 10- to 15-fold following expression of Tax WT while Tax M22 expression led to a modest 2 to 4-fold induction (Figure 3B). For the 1.2 kb galectin-1 promoter construct, expression of TaxWT led to a 10- to 35-fold increase in promoter activity compared to 2 to 6 fold activation when the TaxM22 expression vector was transfected (Figure 3C). These results suggested that the viral protein Tax upregulates transcription from the galectin-1 promoter region, which likely accounts for the observed increase in galectin-1 mRNA levels in both HTLV-I-infected cells and cells transfected with the Tax expression vector.

Lower induction of the galectin-1 promoter by TaxM22, which is deficient for NF-κB activation, raised the possibility that this transcription factor was crucial for Tax-mediated increase in galectin-1 expression. However, Jurkat cells transfected with the 1.2 kb galectin-1 promoter construct did not show higher luciferase activity upon stimulation with two known potent NF-κB activating agents, PMA and TNF-α, thereby strongly suggesting that NF-κB was not involved in the modulation of galectin-1 promoter activity by Tax (Figure 3D). As no known NF-κB-binding sites have been identified from galectin-1 promoter sequence analyses, these results strongly hint on the involvement of a Tax-activated transcription factor different from NF-κB in galectin-1 expression.

### Galectin-1 is more abundant in the supernatant of HTLV-I chronically infected T cell lines than in the supernatant of non-infected cells

**Figure 4:**
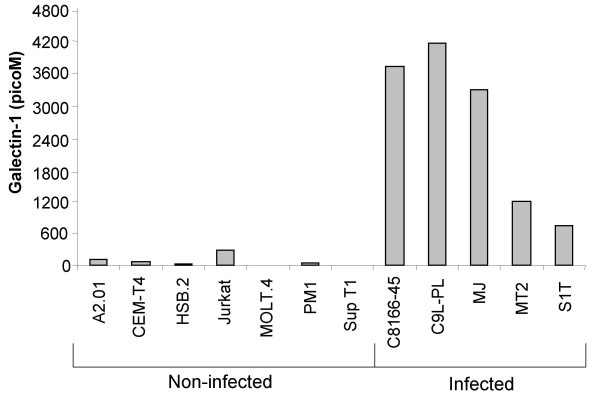
**Comparative analysis of extracellular galectin-1 levels between uninfected and chronically HTLV-I-infected cell lines**. A2.01, CEM-T4, HSB-2, Jurkat E6.1, Molt-4, PM1, Sup T1, C8166-45, C91-PL, MJ, MT2 and S1T cell lines were cultured for 48 hours starting at a concentration of 5 × 10^5 ^cells/ml. The supernatants were then collected, passed through a 0.22 μm filter and analysed for galectin-1 secretion by a galectin-1-specific ELISA as described in Materials and Methods.

As we have demonstrated that HTLV-I-infected cell lines express higher levels of galectin-1 mRNA, we next studied whether these cells produced more extracellular galectin-1. Figure 4 indeed shows that HTLV-I-infected T cell lines released 13 to 50 times higher levels of extracellular galectin-1 than the average level produced by uninfected T cell lines. Interestingly, the S1T T cell line demonstrated the lowest level of extracellular galectin-1 and is known to poorly express Tax.

Together, the data suggest that mRNA and secretion of galectin-1 were both upregulated in cells chronically infected with HTLV-I.

### Galectin-1 increases the infectivity of pseudotyped viruses

**Figure 5:**
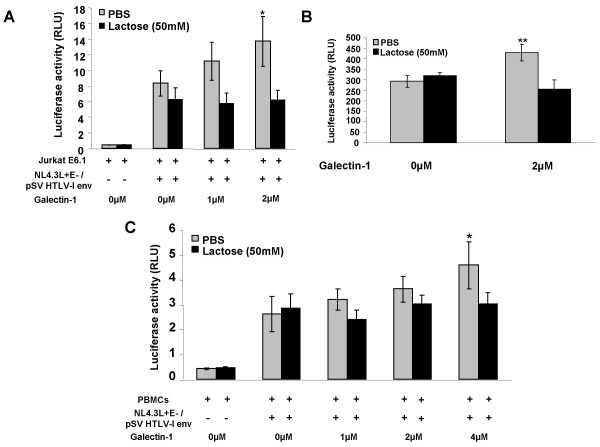
**Soluble galectin-1 positively impacts on the infection of T cell line and PBMCs by HTLV-I-envelope-pseudotyped viruses**. Jurkat cells (**A, B**) or PBMCs (**C**) (1 × 10^5 ^cells) were infected with 10 ng (p24) of HTLV-I envelope-pseudotyped HIV-1 viruses in the presence of different concentrations of purified galectin-1 (0–4 μM), with or without lactose (50 mM). **B**, Jurkat cells were also treated with TNF-α (10 ng/ml). Luciferase activities were measured 48 hours post-infection. The results represent three independent infections and are expressed as the mean luciferase activity value +/- standard deviation (*p < 0.05; **p < 0.01).

As galectin-1 can stabilize cell-to-cell and cell-virus interactions by cross-linking different entities, we studied whether extracellular galectin-1 could facilitate HTLV-I infection. To initiate this study, Jurkat E6.1 cells were first infected with luciferase-expressing HIV virions pseudotyped with the HTLV-I gp46 envelope in the presence of various concentrations of purified galectin-1 (0–4 μM) for 48 hours; luciferase activity was then measured. The use of HTLV-I gp46-pseudotyped virions that can express luciferase allows us to detect a single round of infection and although different from wild-type HTLV-I virions, it should be representative of the type of interactions and fusogenic activities of gp46 occurring on the surface of HTLV-I virions upon infection. Infection of Jurkat E6.1 cells by the pseudotyped virions was increased by 1.6 fold in the presence of 2 μM of galectin-1, an increase which was statistically significant (F = 6.764, p = 0.0138) (Figure 5A). Lactose, an inhibitor of galectin-1, inhibited this galectin-1-promoting effect on HTLV-I infectivity, suggesting that the carbohydrate binding activity of this protein is involved in this increase. In order to increase the luciferase signal, infection of Jurkat cells were also conducted in the presence of the LTR activating agent TNF-α. Results depicted in Figure 5B again demonstrated a highly significant (t = 5, p = 0.0069) positive effect of galectin-1 on infectivity of gp46-pseudotyped virions.

A more physiological model was also used to study the impact of soluble galectin-1 on infection by HTLV-I pseudotyped virus. PBMCs isolated from a healthy donor were stimulated with IL-2 and PHA-L for 72 hours and, after washing, were then similarly treated upon infection by the HTLV-I gp46-pseudotyped virions. The infection of PBMCs by pseudotyped virions was increased by 1.8 fold in the presence of 4 μM of galectin-1 (Figure 5C). The positive modulation on virus infection was determined to be statistically significant (F = 4.364, p = 0.0425).

To eliminate the possibility that galectin-1 was positively modulating LTR activity of the integrated proviral DNA of our gp46-pseudotyped virions, Jurkat cells were transfected with a vector containing the luciferase reporter gene under the control of the HIV-1 LTR, after which different concentrations of galectin-1 (0–4 μM) was added. Measurement of luciferase activity demonstrated that the presence of galectin-1 had no impact on the transcription levels dependent on the HIV-1 LTR (data not shown).

Hence, these results show that extracellular galectin-1 increases infection of a T cell line and PBMCs by free HTLV-I gp46-pseudotyped viruses and that this increase relies on the binding of cell/virus surface carbohydrates by the galectin-1 CRD.

### Effect of galectin-1 on gp46-mediated cell fusion in a co-culture assay

**Figure 6:**
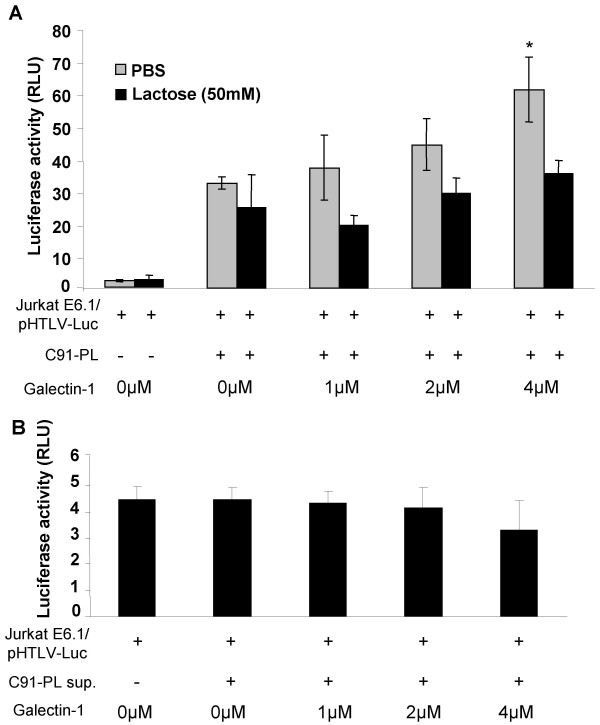
**Soluble galectin-1 increases the extent of HTLV-I LTR activation in co-culture assay**. Jurkat cells were transfected with 15 μg of pHTLV-Luc and cultured for 24 hours. The transfected cells were then incubated for an additional 24 hour with an equal number of HTLV-I-infected C91-PL cells (**A**) or supernatant of C91-PL cells (**B**) in the presence of galectin-1 added at various concentrations with or without lactose (50 mM). The cells were lysed 24 hours after co-culture and luciferase activities were measured. The results represent three independent co-culture assays and are expressed as the mean luciferase activity value +/- standard deviations (*p < 0.05; **p < 0.01).

To study whether galectin-1 can possibly facilitate cell fusion events, a co-culture system allowing a quantitative evaluation of cell fusion by luciferase assay was used [58]. This cell line model provided another useful system to assess the gp46-mediated fusion and was thus used to further confirm the results obtained with the gp46-pseudotyped virions. Our results had previously strongly suggested that this induction of luciferase activity could not be attributed to HTLV-I infection following cell-to-cell contact, but was rather involving cytoplasmic exchange likely mediated by the fusogenic capacity of gp46. Briefly, Jurkat E6.1 cells were transfected with pHTLV-Luc containing the HTLV-I LTR upstream of the luciferase gene and were subsequently co-cultured with the HTLV-I-infected cell line, C91-PL. Cytoplasmic exchange can then be estimated by assessing luciferase activity as Tax present in infected C91-PL cells should, upon cellular fusion, activate HTLV-I LTR activity in transfected Jurkat cells. This assay was thus tested in the presence of different amounts of galectin-1 (0–4 μM) for 24 hours, after which luciferase activity was measured. A dose-dependent (and statistically significant at 4 μM; F = 4.192, p = 0.0466) increase in luciferase activity mediated by galectin-1 was noted (Figure 6A). Again, this induction was lactose-sensitive. Of note, a small but non-significant effect of lactose was apparent in co-cultured cells which were not treated with galectin-1, suggesting a possible impact of endogenous galectin-1 in cell fusion affecting luciferase activity. As a control, supernatant from C91-PL cells incubated in the presence of transfected Jurkat cells did not lead to any significant increase in luciferase activity either in the absence or presence of galectin-1, thereby ruling out the effect of extracellular factors acting on HTLV-I LTR activity (Figure 6B). In addition, although we cannot rule out a contribution in this signal from infection events by HTLV-I particles on Jurkat cells, which would similarly induce luciferase expression, previous experiments have suggested that the first 24-hour time course preferentially involves HTLV-I-driven syncytium formation in the modulation of luciferase assay [58].

These results show that soluble galectin-1 can also increase cytoplasmic cell exchange likely occurring though gp46-dependent cell fusion events between an HTLV-I-infected cells and uninfected T cells, again being inhibited by the addition of lactose.

## Discussion

HTLV-I is a poorly infectious virus and, in this regard, the presence of various molecules that facilitate infection may be important for viral transmission. Several studies have been conducted on the implication of adhesion molecules incorporated by retroviruses (especially for HIV-1) and their positive impact on viral replication [59]. Similar studies have revealed that cell surface adhesion molecules could affect the infection and syncytium formation related to HTLV-I [8,13,60-63]. In addition, certain studies have also indicated that soluble factors were also possible modulators of the HTLV-I infection process [64,65]. Galectins are a family of proteins involved in cell adhesion but few studies have been conducted on their possible involvement in viral infection [66]. In the present study, we have focused on galectin-1, mainly because of its capacity to mediate cell-to-cell contact but also because this protein is expressed by activated T cells and cells from lymphoid tissue, a major site of infection by HTLV-I.

In this study, we have demonstrated that galectin-1 is more strongly expressed and secreted in chronically HTLV-I-infected T cell lines compared to uninfected T cells. These results agree with the study of Pise-Masison and colleagues, which showed through DNA microarray experiments that galectin-1 gene expression is upregulated in HTLV-I-transformed and immortalized cell lines [19]. Furthermore, we have demonstrated that the viral Tax protein could be involved in the upregulation of galectin-1 expression. Generally, Tax directly activates gene transcription by the activation of CREB, NF-κB and/or SRF transcription factor [67]. Using Tax mutants and known NF-κB activators, we have shown that CREB, SRF and NF-κB are not involved in Tax-induced galectin-1 expression. This was surprising given that the Tax mutant TaxM22, which is deficient for the activation of NF-κB, was less efficient in activating galectin-1 expression when compared to wild-type Tax. However, no typical NF-κB binding consensus sequences have been identified in the galectin-1 promoter region tested in this study. In this regard, it should be noted that Tax M22 has been demonstrated to be deficient for the activation of another transcription factor namely NFAT [68,69]. In addition, as the galectin-1 promoter has eight Sp1-potential sites in its 1.2 kbp region, six of which are shared with the 0.5 kbp region, Sp1 might be such a potential transcription factor. Indeed, it has been shown that Tax can interact with Sp1 and the resulting complex is important for the transactivation of PTHrP P3 and GATA3 promoters [70,71]. Alternatively, Tax may indirectly induce galectin-1 expression by maintaining a chronic activation of infected cells. In HTLV-I-infected cells, the constitutive expression of the LTR allows a weak expression of Tax. Since activated T lymphocytes express galectin-1, it may be possible that this chronic activation of HTLV-I-infected cells by Tax indirectly induces galectin-1 expression. Finally, although our results argue for an implication of Tax, it should be stated that other HTLV-I proteins might also participate in the modulation of galectin-1 expression in infected cells as elevated galectin-1 mRNA levels were detected in the S1T cell line, which poorly expresses Tax.

As cell-free virus has very low infectivity, it is assumed that HTLV-I infection is mediated by the interaction between non-infected and infected cells, although recent evidence has demonstrated that cell-free virus can infect isolated dendritic cells [72-74]. We have previously demonstrated that galectin-1 increases HIV-1 infectivity through stabilizing the virus adsorption step [35]. In addition, galectin-1 may mediate cell-cell interaction as galectin-1 can cross-link cells. Using pseudotyped HIV-1 virions, which harbour HTLV-I gp46 envelope protein and express the luciferase reporter gene, we showed that, in both Jurkat and PBMCs, virion infection was significantly promoted in the presence of galectin-1 in a glycan binding-dependent manner, suggesting that galectin-1 increases the infectivity of HTLV-I virus particles in this system. We also used a quantitative system which mimics the mechanism involving gp46-mediated fusion in a cell-to-cell fashion. Again, galectin-1 increased this HTLV-I-induced gp46-mediated cell fusion showing that galectin-1 might also stabilize the interaction between infected and non-infected cells. Of note, no extracellular factors seemed to act upon galectin-1-mediated induction of HTLV-I LTR activity in this co-culture system as judged by results obtained with C91-PL supernatant. However, at this point, it cannot be dismissed that other possible gp46-independent cytoplasmic exchange modulated by galectin-1 might be taking place and lead to this upregulation of luciferase activity. Further experiments will be needed to assess this issue.

Although galectin-1 concentrations in our infection experiments were higher than the measured levels from the supernatant of infected cells, *in vivo *conditions are likely to differ from our cell culture settings. Previous studies have demonstrated that lymphoid organ tissues are sites where an important number of infected T lymphocytes are located [75-77]. Given that these tissues represent a more confined space, the concentration of galectin-1 secreted by surrounding infected cells may represent a more appropriate environment and increase the concentration of galectin-1 to effective levels. Indeed, we have previously reported that the tonsil tissue contains 10 to 20 μM galectin-1 [35]. Moreover, the transmission of HTLV-I to target cells has been shown to require the formation of a virological synapse following a cell-cell contact [7]. This synapse is formed by the binding of host molecules between the HTLV-I-infected T cells and the uninfected T lymphocytes, thereby facilitating virus transmission. Galectin-1 may be concentrated in the vicinity of this virological synapse and more favourably act upon infection. A final issue which needs to be taken into consideration in the current study relates to breast feeding, an important route for HTLV-I transmission. Lactose is an important constituent of breast milk and therefore could be suggested to hinder the action of galectin-1 during this route of HTLV-I transmission. Although one might then argue that galectin-1 is less important for this mode of transmission, it remains to be determined whether high lactose concentrations are also present at sites where initial HTLV-I infection does occur following HTLV-I transmission during breast feeding and where galectin-1 could modulate HTLV-I binding.

## Conclusion

In summary, our study demonstrates a bidirectional interaction between HTLV-I and galectin-1. The data demonstrated that expression of galectin-1 was increased in chronically HTLV-I-infected cells and that this modulation of galectin-1 expression was largely attributed to the viral transactivator Tax in NF-κB- and CREB-independent manners. In addition, this study showed that HTLV-I-infected cells secrete galectin-1 at a higher level than the uninfected cells and that extracellular galectin-1 facilitates HTLV-I infection and promotes higher levels of gp46-dependent cell fusion. Given that our previous studies had demonstrated that HIV-1 is also enhanced in its infectivity by galectin-1 [35,36], other retroviruses (or even other enveloped viruses) could potentially be more infectious in the presence of galectin-1. Further studies will be needed to assess the possible universal action of this β-galactoside-binding protein on the replicative cycle of various pathogens.

## Competing interests

The authors declare that they have no competing interests.

## Authors' contributions

SG carried all RT-PCR analyses, transfection experiments and infection and syncytium formation assay and has drafted the manuscript. IP has conducted the ELISA assay. MO has participated in the design of the study. AV has performed the real-time RT-PCR experiments and has helped in drafting the manuscript. MJT and SS have helped in drafting and finalizing the manuscript and have provided important input on the design of the study. BB conceived the study, participated in its coordination and helped in drafting and finalizing the manuscript.
